# E pluribus unum: we must unify public health in the United States

**DOI:** 10.1093/pubmed/fdac102

**Published:** 2022-11-21

**Authors:** Heather Krasna

**Affiliations:** Department of Health Policy & Management, Columbia University Mailman School of Public Health, New York, NY 10032, USA

Public health professionals have often viewed themselves as invisible heroes, working diligently behind the scenes to save lives. Due to the Covid-19 pandemic, public health has suddenly been put in the spotlight, and the public health system in the United States has been shown to be underfunded,^1^ understaffed^2^ and disempowered^3^ due to decades of underinvestment^4^^(p19)^. One reason for this lack of investment, visibility and power is that the public health workforce in the US is decentralized to the point of ‘disarray’.^5^ The public health workforce is also so diverse, it is difficult to define or enumerate, and does not speak with a unified voice. Because of this disunity, a vicious circle is created: the many splintered voices of public health departments and professional societies may compete with one another for attention and resources; the public, and elected officials who make funding and policy decisions, therefore do not hear clear messaging about the importance of the public health workforce or health departments; other, more unified and better-funded voices push public health priorities aside; and the public health infrastructure therefore continues to be underfunded, understaffed, disempowered and invisible.

## Not one public health system, but 3000

In the United States, the public health workforce is often defined as those individuals who work in government health departments, unlike in the United Kingdom, where the Centre for Workforce Intelligence describes a ‘core’ and ‘wider’ workforce, which includes many sectors.^6–8^ However, even within this narrow definition, the US governmental public health workforce might actually be described as ‘workforces,’ because this workforce is siloed across 2800 local health departments,^9^ > 60 state and territorial health departments,^10^ numerous Tribal governments^11^ and several distinct, often uncoordinated or competing federal agencies.^12^ Unlike in the UK, there is no central registry of public health professionals. This decentralization means each health department faces its own local or state-level regulations, creates its own data and has to expend time and resources to ensure collaboration, especially for larger scale responses to public health disasters.^13^ With no central registry, it is also difficult even to know the number of staff who work in health departments in the USA^14^^,^^15^; without this information, it is difficult to know how much funding the system needs to perform its essential functions.

## Not one profession, but 73

The workforce also lacks a unified voice because it is diverse in terms of the variety of professions or occupations in the field. One of the largest studies of the government workforce includes a taxonomy of 73 different occupations,^16^^,^^17^ and a total of > 90 different occupations were found when analyzing job postings from employers seeking individuals with Master’s level degrees in public health.^18^ Several of these occupations have strong, unique professional identities of their own, including their own professional societies and certifications,^19–21^ or their own defined, unique lists of competencies,^22–27^ such as those established for public health nurses,^22^^,^^23^ epidemiologists,^24^ disease intervention specialists,^21^ community health workers,^25^ laboratory scientists^26^ and health educators.^28^ However, each of these professional societies are much smaller and less powerful than, for instance, the American Medical Association (AMA), which represents physicians and is one of the largest lobbying groups in the United States. And although there are field-wide associations, like the American Public Health Association (APHA), they are far smaller than clinical professional associations; the APHA’s annual revenue is around $16.7 million per year, whereas the AMA’s is $364 million.^29^ And even accounting for their smaller size, public health associations do not conduct as much lobbying or advocacy as healthcare organizations.^30^

The lack of a strong, unified voice means that the public health workforce is less able to defend itself from attack—and the workforce has been significantly under attack in the United States, including harassment of staff and leadership.^31^ With a lack of understanding or support for the role of public health experts, many localities have stripped legislative and regulatory powers from public health authorities.^32^ And although the Covid-19 pandemic has caused the US to spend around $3.74 trillion on COVID-19 relief,^33^ this public health crisis has still failed to translate into sustained investment in the public health workforce. The American Rescue Plan Act (ARPA) sets aside only $7.4 billion for public health workforce investment—and this amount is temporary, to be spent over 5 years.^34^ With research showing 80 000 new hires are needed to replenish the workforce,^35^ many billions of dollars would be needed every year, permanently.

## Unifying the professions

How can we unify and empower the field, when it is so diverse and disempowered?

There are new calls, including from the Commonwealth Fund’s Commission on a National Public Health System, to create a more unified public health system at a structural level.^36^ Such efforts could benefit the public health workforce over the longer term.

In the shorter term, one option is to raise awareness of public health as its own profession, with a professional identity as powerful as that held by physicians. First, academic institutions of public health might begin to establish this identity by incorporating an oath and symbol in their educational programs—similarly to how physicians take an oath, and have symbols of their profession like the caduceus. Second, there are currently ~124 000 public health students in the United States, with 35 000 graduating each year, yet there is no association specifically representing public health students. An active public health students’ and graduates’ association could magnify the advocacy efforts of existing public health associations, especially around increasing funding for the workforce and protection from attacks on the workforce and its authority. Third, a more deliberate linkage between academic institutions of public health and the governmental public health workforce would strengthen the power of the workforce, whereas ensuring academia fulfills its role of preparing public health professionals.^37^ Fourth, new efforts to enumerate and research the workforce can gather the data needed to illustrate workforce gaps.^38^ Fifth, public health professionals who are members of professional societies or associations can encourage their societies to invest in more powerful advocacy efforts, and to work together in coalition, to further strengthen the voice of public health.^30^

Another approach is to raise public awareness of, and appreciation for, the public health workforce through marketing and awareness-raising efforts. The ARPA funding to invest in the workforce is meant to begin solving the staffing shortage and could increase public awareness of the field.^34^ However, even with this new funding, there could still be difficulty in recruiting new workers into the field due to its decentralization.^39^ Unlike the UK’s National Health Service, which maintains a unified applicant tracking system for individuals interested in joining,^40^ the United States not only has 2800+ different health departments, but many of these agencies have complex civil service hiring mechanisms that differ from one local health department to the next, even in the same state. New efforts are currently underway via the Association of State and Territorial Health Officials to create an attractive recruitment website which will connect job-seekers to the thousands of health departments in the US, and to educate the public about what public health workers do; this may unify the image of the workforce and facilitate replenishing its numbers.

The United States was created through unifying 13 disparate colonies and our national motto is *e pluribus unum,* ‘from many, one*.*’ The public health workforce must come together to achieve the power it needs to protect the public’s health.
